# Why did Professor Per‐Ingvar Brånemark never receive the Nobel Prize in Medicine?

**DOI:** 10.1002/cre2.72

**Published:** 2017-07-03

**Authors:** Asbjørn Jokstad

**Affiliations:** ^1^ CEDR UK

Each fall, invitations are sent out to some two 3,000 academic scholars to nominate a worthy candidate for the Nobel Prize in Physiology or Medicine. An intricate rotation pattern established by the Nobel Committees assures that different scholars receive invitations each year. In 2006, a university colleague in Belgium received this invitation and embarked on gaining support for nominating Professor Per‐Ingvar Brånemark from Gothenburg, Sweden, for the Nobel Prize in Physiology or Medicine. The FDI World Dental Federation president at the time was Michele Aerden, also from Belgium, and she strongly endorsed the initiative. At the time, I was contracted as the Scientific Affairs manager of FDI, so I was tasked to work out a sound justification for nominating Professor Per‐Ingvar Brånemark. Unfortunately, he did not receive the prize in 2006. Nor did he received the physics prize in 2004, which was the blueprint for our nomination.

Just before Christmas, I received an invitation to nominate a candidate for the Nobel Prize in Physiology or Medicine. The email evoked fond memories, and I would not have hesitated to nominate once again Professor Per‐Ingvar Brånemark for his role in the discovery of the biological process that we today call osseointegration. However, a sad fact is that he passed away in December 2014, and the Nobel Prize cannot be awarded posthumously.

According to the will of Alfred Nobel, it is the scholars employed at the Karolinska Institute in Stockholm, Sweden, who shall decide who will receive the prize. The essential criterion is that the prize should be awarded to the individual who has brought the greatest benefit to humankind the year before, alternatively that the realization of the impact of the discovery has been made the year before. It is hard to imagine that anyone can discount that the contributions from the research team directed by Professor Per‐Ingvar Brånemark has benefitted humankind. Moreover, the increasing number of individuals around the globe reinforces its impact each year not by thousands, but by millions who have obtained better health and improved quality of life because of surgically placed alloplastic implants. Few other discoveries in medicine can match this phenomenal impact. It is therefore intriguing to speculate why Professor Per‐Ingvar Brånemark never received the Nobel Prize. Perhaps one misconception is what actually the Brånemark research team in Gothenburg, Sweden, contributed to humankind.

The Brånemark research team was not the first to propose that titanium seemed to be a suitable material for implantation. A research team in Great Britain proposed this already before WWII (Bothe, Beaton, & Davenport, 1940), and a researcher in the United States endorsed the idea after the war years (Leventhal, 1951). The first researchers reported a “*tendency for the bone to fuse with titanium*,” and the second “*bone becomes attached to titanium … since titanium adhers to bone, it may prove to be an ideal metal for prostheses*.” Subsequent research revealed that titanium had a low corrosion rate in chloride environments and a lack of toxicity of the titanium salts. Biological researchers in dentistry (Beder & Ploger, 1959) and in medicine, including Hans Emnéus (Emnéus & Stenram, 1960) who was one of the mentors of Professor Per‐Ingvar Brånemark, showed that bone tolerated very well implanted pieces of titanium. A simple search for titanium on PubMed generates some 350 papers published before December 31, 1970. A close scrutiny of the papers that focus on titanium and tissue interactions does not reveal any data that cast more light on how and why “*bone become attached to titanium*.” That is, until the detailed description of *the interface phenomena between titanium and bone under different experimental conditions* in a publication in 1969 (Brånemark et al., 1969). The authors justified the experiments by prior knowledge gained in prior studies on nutritive capillaries in bone marrow (Brånemark et al., 1964). The publication was recognized in an influential review of dental implants presented by The Council on Dental Materials and Devices of the American Dental Association in 1972 (ADA Council on Dental Materials and Devices, 1972). It is remarkable that the reviewers did not emphasize or discussed the study more in detail, since it was the only amongst the 274 references that reported promising experimental data with cylindrical endosseous titanium implants. Perhaps it can be explained by a skepticism voiced by orthopedic engineers a few years to use an alloplast with a relatively low elasticity modulus and thus an inherent risk of interfacial stress, at least when considering blade implants (Anonymous, 1980).

However, the very capable members of the Brånemark research team and likely also some competent readers grasped the significance and possible opportunities for clinical improvements enabled by the better understanding of the osseointegration phenomenon. Multiple research and development teams can share the credit for the many *innovations* in clinical practices and improved designs of endosseous oro–craniofacial implants enabled by the new knowledge. Even Professor Per‐Ingvar Brånemark and his team proceeded to translate the research to medical innovations, and their detailed efforts were described in a seminal publication from 1977 (Brånemark et al., 1977) and further elaborated in another important publication in 1981 (Albrektsson, Brånemark, Hansson, & Lindström, 1981). For the interested, detailed recounts of both the basic and clinical research are presented in “The Osseointegration Book” that was published in 2005 (Brånemark, 2005).

Attributing the discovery of “(modern) titanium implants” to Professor Per‐Ingvar Brånemark is a contentious claim. He entered the commercial world after having licensed the manufacturing rights to a Swedish company and filed a patent in Sweden and in the United States in 1979 (www.google.com/patents/US4330891), actually some months after Straumann had filed their patent in the United States for a dental implant fabricated in titanium (US4180910). However, already eight patents had by then been filed for dental implants made out of titanium or a titanium alloy (US3579831, US3590485, US3605123, US3729825, US3829972, US3849888, US3851393, US4051598, and US4060896). Even though Professor Per‐Ingvar Brånemark lent his name to a brand of dental implants manufactured by one particular company, I believe that it is not for these implants that he ought to be recognized and remembered, but rather for his *discovery of osseointegration phenomenon*.

I do not believe there has ever been a conspiracy to not offer the Nobel Prize to Professor Per‐Ingvar Brånemark for any particular reason. My speculation is whether the Nobel secretariat and perhaps some researchers and colleagues today never realized the uniqueness of the *discovery of osseointegration phenomenon*. Multiple creative competent research and development teams may on the other hand share the credit for the *innovations* that followed because of this discovery. My theory has been fueled by attending recent meetings in implant organizations where I have listened to current researchers offering historical recounts of the Swedish, Swiss, German, and North America implant stories. The synopsis of these narratives is that research groups in each country seemingly independently developed titanium implants in the 70s more or less oblivious of the groundbreaking work by Professor Per‐Ingvar Brånemark and his research team. I am not entirely persuaded of this theory for several reasons. At least one lecturer offered the explanation that the Brånemark research team published their findings in “a small and obscure Scandinavian journal,” and they remained therefore unknown to the international research community. The claim can be believed if it had not been for the fact that the comprehensive ADA review on dental implants from 1972 that is cited above identified the Brånemark et al. paper from 1969 (ref. # 255) reporting successful integration of titanium implants in animal experiments, in contrast to the 22 other animal studies presented in table 6. It seems incomprehensible that nobody within the pertinent research field would explore how this was possible and how it could be achieved.

In my opinion, there is no doubt that Professor Per‐Ingvar Brånemark both qualified and deserved a Nobel Prize in Medicine. This should not be interpreted as a criticism of the decision makers in the Nobel Committee at the Karolinska Institute, since their lists of worthy candidates are long and impressive (http://www.nobelprize.org/nomination/archive). We will likely never learn their rationale for electing the annual Nobel Prize winners, because minutes from the committee deliberations are not public information. The same applies to the qualities of the evaluation reports based on the “detective work” undertaken by the Nobel secretariat, whose task is to check the veracity of the claims detailed in the numerous nominations that they receive. One may ask whether it really matters to whom the decision committee at the Karolinska Institute in Stockholm, Sweden, award the Nobel Prize. Professor Per‐Ingvar Brånemark was likely nominated multiple times, and for those who hang around the names of the nominees and other information about the nominations will be revealed 50 years from now. We remain all indebted to him and his research team for the research on osseointegration and alloplastic biomaterials conducted over the near five decades in Gothenburg, Sweden, and elsewhere.
